# Meteorological Register

**Published:** 1835-07-01

**Authors:** 


### METEOROLOGICAL REGISTER,

FROM MARCH 1, 1834, TO MAY 31, 1835.

By Messrs. Harris and Co., Mathematical Instrument Makers, 50, High Holborn.

| | Thermometer. | | Barometer. | | De Luc's Hygrometer | | Winds. | | Atmospheric Variations. | | |
|---|---|---|---|---|---|---|---|---|---|---|---|
| | *max.* | *min.* | 9 *a.m.* | 10 *p.m.* | 9*a.m.* | 10.*pm.* | 9 *a.m.* | 10 *p.m.* | 9 *a.m.* | 2 *p.m.* | 10 *p.m.* |
| Mar. 1 to 7 | 52 | 34 | 29.87 | 28.85 | 58 | 51 | SW | WSW | snow | fine | rain |
| 7 to 14 | 53 | 35 | .98 | .85 | 60 | 51 | SSW | WSW | cloudy | rain | fine |
| 14 to 21 | 54 | 36 | 30.06 | 29.53 | 60 | 53 | N | W | rain | rain | fine |
| 21 to 31 | 54 | 35 | .22 | .53 | 61 | 50 | NNE | WNW | cloudy | fine | rain |
| Apr. 1 to 7 | 65 | 41 | .14 | .51 | 62 | 50 | SSE | SSW | rain | rain | fine |
| 7 to 14 | 63 | 40 | .16 | .67 | 59 | 44 | NNE | SSW | fine | fine | fine |
| 14 to 21 | 58 | 30 | .20 | .70 | 49 | 44 | NW | W | fine | snow | rain |
| 21 to 30 | 59 | 35 | .20 | .25 | 67 | 46 | NNW | NE | rain | snow | fine |
| May 1 to 7 | 61 | 35 | 29.86 | .36 | 68 | 50 | SSW | WSW | rain | fine | rain |
| 7 to 14 | 68 | 44 | .80 | .31 | 70 | 49 | SW | SE | fine | rain | rain |
| 14 to 21 | 70 | 45 | .94 | .33 | 70 | 45 | E | NNE | rain | fine | cloudy |
| 21 to 31 | 63 | 40 | .83 | .06 | 62 | 44 | WSW | NNW | rain | fine | cloudy |

The quantity of Rain fallen in March, was 2 inches.

April, was 96 100ths of an inch.

May, was 1 inch and 78 100ths.

